# Bone Regeneration After Nail Distraction Osteogenesis: Review of Current Knowledge and Application Combined with a Case Report with Radiological, Histological, and Electron Microscopic Analysis

**DOI:** 10.3390/jcm13216504

**Published:** 2024-10-30

**Authors:** Nader Maai, Florian A. Frank, Thomas A. Schildhauer, Matthias Königshausen

**Affiliations:** 1 Department of General and Trauma Surgery, BG University Hospital Bergmannsheil, Ruhr University Bochum, 44789 Bochum, Germany; thomas.a.schildhauer@rub.de (T.A.S.); matthias.koenigshausen@bergmannsheil.de (M.K.); 2 Center for Musculoskeletal Infections (ZMSI), University Hospital Basel, 4031 Basel, Switzerland; florian.frank@usb.ch; 3 Department of Orthopedic and Trauma Surgery, University Hospital Basel, 4031 Basel, Switzerland

**Keywords:** limb lengthening, distraction osteogenesis, nail, bone regeneration, bone morphology

## Abstract

**Background**: Limb-lengthening surgeries via nail distraction osteogenesis (DO) have become more popular lately. This provides an opportunity to study human bone that has grown longer. **Case details**: We present a case of a 22-year-old male who underwent internal upper and lower leg lengthening by 12 cm and 6 cm, respectively, under full weight bearing. He requested bilateral femoral shortening by 4 cm using a shortening nail, 24 months after the index surgery. The regenerated bones were harvested and analyzed. **Results**: Good bone quality and well-organized structure were observed in the regenerated bones compared with the native human adult bony architecture. **Conclusions**: This case demonstrates that bilateral bone regeneration during DO with a nail can result in a bone morphology that is comparable to that of native adult human bony macro- and micro-anatomy. This supports the effectiveness and potential of this surgical approach for limb lengthening and shortening procedures, although more investigations are necessary in this regard.

## 1. Introduction

The concept of limb lengthening was introduced first by Langenbeck in 1869, who suggested that axial traction stimulates bone growth [1]. The first documented lengthening procedure was performed in 1905 by Codivilla, which involved an osteotomy followed by axial traction and immobilization in a Thomas splint [2].

The development of external fixators, which allowed controlled distraction and the animal and biomechanical research conducted by Gavril Ilizarov, significantly improved the technique and understanding of distraction osteogenesis (DO) [3,4]. However, it is important to note that external fixators can be associated with high complication rates, including pin site infection, deep sepsis [5]. To overcome these issues internal limb lengthening devices have been developed. The first reported internal lengthening device was invented by Alexander Bliskunov in 1983 [6]. Over time, many different lengthening nails have been developed [7,8,9,10,11,12].

As described by Codivilla [2], DO consists of three phases: latency, distraction, and consolidation. In the first phase, latency, which usually lasts for 5–7 days after osteotomy, the bone segments are stabilized in accordance with Ilizarov’s recommendations [4]. This period is characterized by a series of complex biological events. These events eventually return to their initial levels [13].

The subsequent distraction phase involves the controlled application of mechanical tension at a rate of 1.0–1.5 mm per day and a frequency of 2–4 times per day until the desired lengthening is achieved [4]. This period initiates a series of cellular and molecular events that support intramembranous ossification and promote cell differentiation [13,14]. It also allows for the resorption of newly formed mineralized cartilage [13].

Distraction stress induces many cellular and molecular responses that stimulate the formation of new bone [15]. The influence of mechanical tension on increasing osteoblast activity and encouraging bone marrow mesenchymal stem cells (BMSCs) to become osteoblasts has been well documented [16,17]. In addition, the tension created by distraction has a major impact on the process of creating new bone tissue [18,19,20,21,22,23].

The consolidation phase, which usually lasts from six months to a year or more, is characterized by a long period of immobilization. During this phase, the distracted callus grows with the mechanical assistance of the fixation device, preventing cartilage formation to ensure stability [24]. Bone remodeling begins in the consolidation phase. This is characterized by the formation of lamellar bone with elements such as bone marrow, which provides mechanical support for a prolonged period [25,26]. The biological processes in the consolidation phase involve the joining of bone columns, the recruitment of osteoclasts, and shape change (remodeling) [25,26,27,28,29,30].

Used primarily to correct jaw deformities where bone lengthening is indicated, DO is a relatively new concept in maxillofacial surgery. Initial investigations have focused on the nature and process of DO, particularly in the mandibular region, where particular complications are anticipated because of the axial force. Whilst the literature has reported successful DO using toothborne devices, it has been shown that the quality and structure of the newly formed bone may differ due to different activation protocols being used [31]. For example, the quality and structure of newly formed bone may be adversely affected by reducing the frequency of activations from once to twice daily, leading to instability.

DO is biologically similar to fracture healing, with specific features in the distraction phase itself, as shown by histological and radiological examination [32]. During this phase, a large number of interleukins and growth factors are expressed, attracting mesenchymal stem cells that then differentiate into osteoblasts and other cells, resulting in callus formation [32]. As distraction continues, five characteristic zones can be distinguished within the distraction gap. These include unmineralized bone in the center, remodeling bone at the periphery, and mineralizing bone in between [32].

Further research has shown molecular, histology and radiology parameters remain important characteristics affecting bone regeneration after DO [33,34,35,36]. For example, the microstructural appearance of newly formed bone after distraction osteogenesis differs from that of native bone. Its high degree of anisotropy appears to be maintained in association with the direction of the distraction vector [33]. Its quality—the bone that has formed after DO—may be adequate, but less mineralized, with a less dense pattern in the trabeculae when compared to native bone [34]. BMPs may be critical mediators of bone formation in craniofacial DO, as they were highly expressed during mandibular DO. The expression of BMPs suggests a temporal relationship with bone formation and a spatial relationship with bone formation during DO.

The key to optimizing treatment outcomes in maxillofacial surgery will be to understand the mechanisms of DO and the modulating variables [35].

Animal models were also developed for studying bone regeneration after DO and provided valuable insight into this procedure [36].

For example, an original experimental model of mandibular DO in edentulous rats has been presented in which the quantity and quality of the intramembranous bone were sufficient. This model may be useful for the study of bone regeneration and the optimization of DO techniques [36].

Focusing on DO in limbs, Li et al. [23] reviewed the current methods for promoting bone formation and consolidation in DO, with special emphasis on biometrics. They found that several methods, such as physical therapy, gene therapy, growth factor-based therapy, stem cell-based therapy, and improved distraction devices, have been used to reduce the time required for DO treatment with some degree of success. However, none of these methods are widely accepted in clinical practice for reasons such as high cost, short biological half-life, and ineffective delivery routes. Finally, the authors concluded that there are encouraging potential clinical applications of magnesium-based devices to accelerate DO.

Recent developments in deformity correction with a special focus on bone lengthening were reviewed by Barakat et al. [24]. They presented case examples of the use of lengthening nails to treat complicated malunions and non-unions, as well as a new approach to bone transport. They found that new methods of intramedullary bone lengthening were replacing the old-fashioned external frame in conjunction with deformity correction. The authors concluded by saying that because of the great advances in intramedullary lengthening devices—which have fewer complications and make patients happier—it may be that we are seeing the end of the age of the circular frame.

Degen et al. [25] investigated pain levels during DO with LN in a large sample. They found that pain levels in the distraction phase are typically low, steadily decreasing, and can be well managed with mostly non-opioid analgesics. They noticed an interesting change in pain perception in children as they moved from the distraction phase to the consolidation phase. It is possible that their response to discomfort is significantly reduced during this later phase due to limited mobility.

Dvorzhinskiy et al. [26] compared hospital, surgeon, and total costs between lengthening then nailing (LATN) and magnetic lengthening nail (MLN) for tibial lengthening. They found that the overall costs of LATN and MLN were similar in patients undergoing tibial lengthening. However, the MLN required fewer procedures but took longer to set than the LATN.

Richardson et al. [27] made a comparison between femoral lengthening by lengthening over a nail (LON) and the MLN method. They found that although the cost of implants was higher for MLN than LON, this appeared to be offset by the lower number of procedures required. In general, the two procedures had almost identical total costs, but MLN showed a reduced number of procedures and a faster union time.

Frommer et al. [28] conducted a study on femoral lengthening using magnetically driven antegrade intramedullary lengthening nails. They concluded that this method appeared to be accurate and reliable for femoral lengthening. However, they also stated that, depending on the cause, there may be a risk of unplanned additional surgery, and many patients experience temporary joint stiffness.

The use of extramedullary placement of a magnetic expandable intramedullary nail for femoral lengthening in skeletally immature patients was reviewed by Dahl et al. [29]. They found that this method, with complication rates similar to other femoral lengthening methods, can achieve lengthening without the need for external fixation. The authors emphasized the need for careful planning of the length of the implant, protection against subluxation of the knee during the lengthening process, and minimization of deformity in the regenerating tissue. They concluded by considering the off-label use of these devices outside of the bone marrow to reduce the difficulties of external fixation in young children. They also said that there is a need for future all-internal technology specifically designed for safe limb lengthening in this age group.

The work of Merchan et al. [30] summarises the literature on the PRECICE nail (NuVasive), its use, design, and safety for intercalary reconstruction after tumor removal. They found that bone transport with the PRECICE nail is a viable alternative to Ilizarov distraction and has the advantage of not requiring an external fixator. In large defects, the use of the PRECICE nail can be combined with a locking plate to increase stability and preserve limb length. In the authors’ opinion, while DO is the least-used choice for intercalary reconstruction, it can have the best outcomes for patients.

The quality of bone healing after DO was investigated in studies by Jochymek et al. [37,38] and Han et al. [39]. Although these studies were quite different in design and focus, there were similarities. Jochymek et al. reported that satisfactory bone healing was achieved in the majority of cases. However, poor bone healing was associated with older patient age and lack of visible periosteal callus 5 weeks after surgery. This has been confirmed by Han et al., who showed that the quality of bone healing varies with respect to the period of consolidation after distraction osteogenesis.

A similarity among the studies is the observation that DO bone healing is affected by different factors. For example, Jochymek et al. [37,38] identified age and periosteal callus as the most influential factors, while Han et al. [39] found that soft tissue overlying the distraction osteogenesis area was important for bone formation. The conclusion from these findings is that DO is complex and various factors should be taken into account for optimal bone healing.

However, there were also some differences in the results of some of these studies. While the study by Jochymek et al. [37,38] focused on the clinical aspects of DO in patients, an experimental study in animals was conducted by Han et al. [39] to investigate the histomorphological changes of tubular bone after widening distraction osteogenesis. Therefore, the results and implications of the studies were different. While the studies by Jochymek et al. [37,38] provided information on the clinical management of DO, Han et al. [39] provided information on the biological process of bone healing after DO.

Electron microscopy, particularly scanning electron microscopy (SEM), offers even higher resolution for ultrastructural analysis of regenerated bone. SEM allows for detailed visualization of surface topography, pore size, and interconnectivity in newly formed bone—key factors in determining its mechanical and biological properties [28,31]. Additionally, the elemental composition of the regenerated bone and the calcium-to-phosphorus (Ca/P) ratio, a marker of bone mineralization, can be assessed using a combination of SEM and energy-dispersive X-ray spectroscopy (EDS) [40].

Despite the potential of these techniques to reveal the characteristics of regenerated bone following nail distraction osteogenesis, very few studies have thoroughly examined the radiological, histological, and ultrastructural features of newly formed bone in a clinical context. Our case report and narrative review aim to address this gap by providing a detailed analysis of the regenerated bone in a patient who underwent bilateral femoral and tibial lengthening using an intramedullary nail system. A secondary objective is to offer insights into the quality and nature of regenerated bone, which may inform future research and clinical decision-making in limb-lengthening surgery.

## 2. Case Report

**Figure 1:**
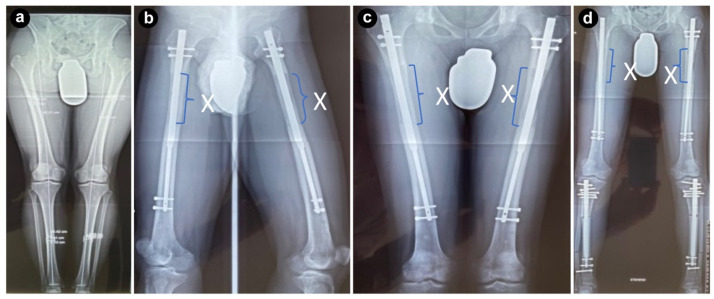
X-rays during each surgical step. X indicating the lengthening area: (**a**) X-ray prior to the initial surgery, (**b**) during upper leg lengthening, (**c**) finished upper leg lengthening, and (**d**) after removal of irritating proximal screws right upper leg and beginning of lower leg lengthening.

A 22-year-old Asian male, dissatisfied with his height since adolescence, had a long-standing desire to increase his stature. At the time of presentation, he was 163.5 cm tall, weighed 62 kg, and had a wingspan of 166 cm. Radiological examination revealed femoral lengths of 422.1 mm on the right and 422.6 mm on the left, and tibial lengths of 354.2 mm on the right and 353.2 mm on the left. His target height was between 178 and 180 cm (Figure 1a). With no history of metabolic disorders and no ongoing treatments that could interfere with bone regeneration or surgery, he was considered a suitable candidate for a limb-lengthening procedure.

### 2.1. Preoperative Considerations and Decision-Making

Extensive consultations with orthopedic specialists, along with detailed discussions about the potential risks and benefits, ultimately led the patient to opt for surgery. These discussions included a thorough explanation of the procedure, recovery timelines, possible complications, and the impact on daily life and mobility before and after the surgery. With no contraindications and the patient’s strong desire for height increase, the decision was made to proceed with the surgery.

### 2.2. Surgical Interventions

#### Femoral Lengthening (February 2019)

In February 2019, the patient underwent bilateral femoral lengthening using an intramedullary nail system (Medi-Tech GmbH, Betz Institute, Wadern, Germany). The initial lengthening was set at 0.6 cm, followed by gradual lengthening at a controlled rate of 1 mm per day through rotational movements of the upper leg. Over time, both femurs were lengthened by a total of 12.6 cm. This gradual process allowed the bone and surrounding tissues to adapt, reducing the risk of complications such as nerve damage or muscle contracture.

### 2.3. Early Postoperative Care

Intensive physiotherapy was initiated to ensure proper healing of both soft tissues and bone structures, helping them regain strength and proper alignment. The patient was allowed to bear full weight during the lengthening phase, which contributed to the healing process. Six months post-surgery, the patient had regained a normal gait with full weight-bearing capacity. The only complication was irritation caused by a proximal screw in the right upper leg, which was removed after bone consolidation (Figure 1b–d).

#### Tibial Lengthening (June 2020)

**Figure 2:**
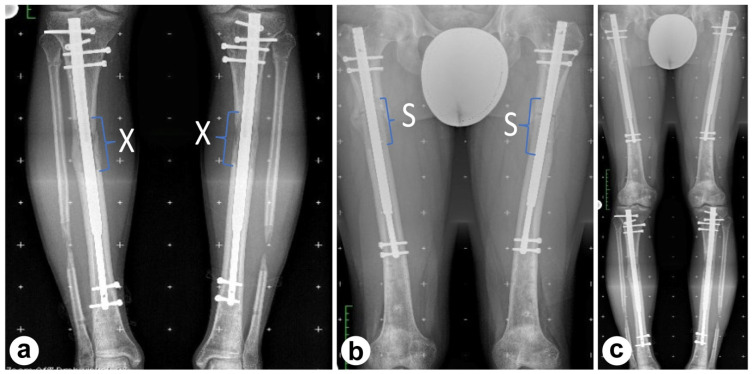
X-rays during third stage of procedure, X indicating the lengthening area: (**a**) X-ray upon completion of upper leg lengthening, (**b**) X-ray of both upper legs during cosmetic shortening procedure, S marking the shortened bone, and (**c**) long leg view of completed lower leg lengthening and completed upper leg lengthening.

In June 2020, the patient underwent a second surgery for tibial lengthening. Using the same intramedullary lengthening system, a total of 6 cm was added to both tibiae over a five-month period. Throughout this time, the patient maintained full weight-bearing, which facilitated recovery. Close monitoring ensured that nerve or soft tissue complications were avoided, and within five months, the patient had a well-formed, physiological gait. By the end of this procedure, the patient’s height had reached 182.1 cm, achieving his desired height (Figure 2a).

### 2.4. Post-Lengthening Adjustments and Femoral Shortening

During the tibial lengthening process, the patient became dissatisfied with the relative length of his upper legs compared to his lower legs, seeking more symmetrical proportions. He requested a 6 cm reduction in his upper leg length. Initially, the surgical team was hesitant due to the complexity of reversing the previous procedure, but after thorough discussions and a psychological assessment to ensure the patient’s expectations were realistic, the team agreed to perform the femoral shortening.

#### Femoral Shortening (January 2021)

In January 2021, the initial intramedullary lengthening nails were replaced with specialized shortening nails (Medi-Tech GmbH, Betz Institute). The procedure involved removing a 4 cm segment of the femur by cutting the previously lengthened area and securing the shortening nail, creating a 3 cm gap between the proximal and distal bone fragments.

The shortening began with the right leg and continued with the left 10 days later. Controlled 5 mm increments per day were applied to ensure proper anatomical alignment and maintain soft tissue tension. Analgesics were administered to enable pain-free mobility during the healing period. The shortening procedure was deemed complete once radiological evidence confirmed the closure of the bone gap. This gradual approach helped maintain soft tissue tension and minimized the risk of muscle contracture (Figure 2b,c).

### 2.5. Further Course

The patient tolerated the procedure well and was able to mobilize effectively with full weight-bearing. Since relocating abroad, he has not provided additional radiological follow-up images. However, he reports being pain-free, actively engaged in sports, and highly satisfied with the surgical outcome, with no physical issues noted. In a recent phone conversation, he mentioned plans to have the metal hardware removed within the next two years.

## 3. Materials and Methods

After obtaining the patient’s written informed consent, a histopathological examination of both specimens was performed. In a bilateral operation, two bone segments were resected from the right and left femurs using an intramedullary bone saw.

The first preserved specimen was 43 mm long and had a diameter of 30 mm. The second specimen was 35 mm long and had a diameter of 25 mm. The wall thickness ranged from 3 to 8 mm.

The bone segments were preserved in formalin. Histological examination of the segments was performed first. Following this, the segments were examined using an electron microscope. In addition to this, an examination by means of a high-resolution computed tomography using a Siemens Somatom Definition Edge (120 kV, 168 mAs, 0.3 mm slice thickness; Siemens Healthineers AG, Munich, Germany) was performed.

## 4. Results

After obtaining the patient’s written informed consent, bone specimens from both femurs were collected for histopathological analysis. During the bilateral surgery, bone segments were excised from the previously lengthened areas using an intramedullary bone saw. The specimens measured 43 mm in length with a diameter of 30 mm, and 35 mm in length with a diameter of 25 mm, respectively, with wall thicknesses ranging from 3 to 8 mm. The bone samples were preserved in formalin and underwent histological and electron microscopic examination, as well as high-resolution computed tomography (HRCT) scans using a Siemens Somatom Definition Edge (120 kV, 168 mAs, 0.3 mm slice thickness; Siemens Healthineers AG, Germany).

### 4.1. Results of Bone Analysis

#### 4.1.1. CT Scan Results

**Figure 3:**
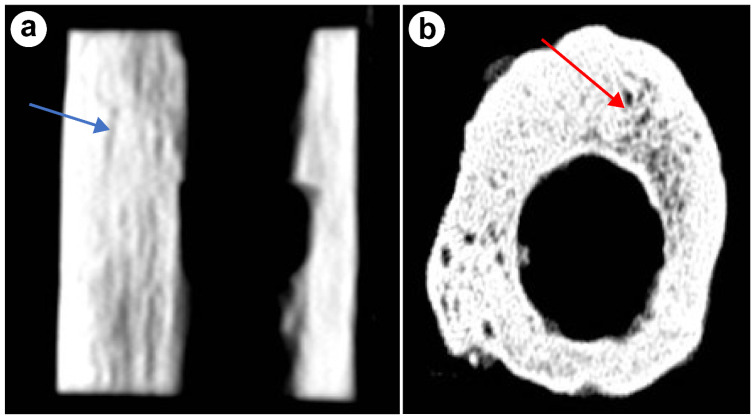
(**a**) CT scan illustrating the trabeculae (blue arrow), (**b**) CT scan revealing bone canaliculi (red arrow) and marrow hole.

HRCT revealed well-organized lamellar bone with dense cortical layers and distinct marrow spaces. The trabeculae were aligned with the loading axis of the bone, reflecting physiological adaptation to the lengthening process. Canaliculi, responsible for the transport of nutrients and waste, were clearly visible. Bone density averaged 1128 HU and 1247 HU, indicating good mineralization (Figure 3a,b).

#### 4.1.2. Histological Examination

**Figure 4:**
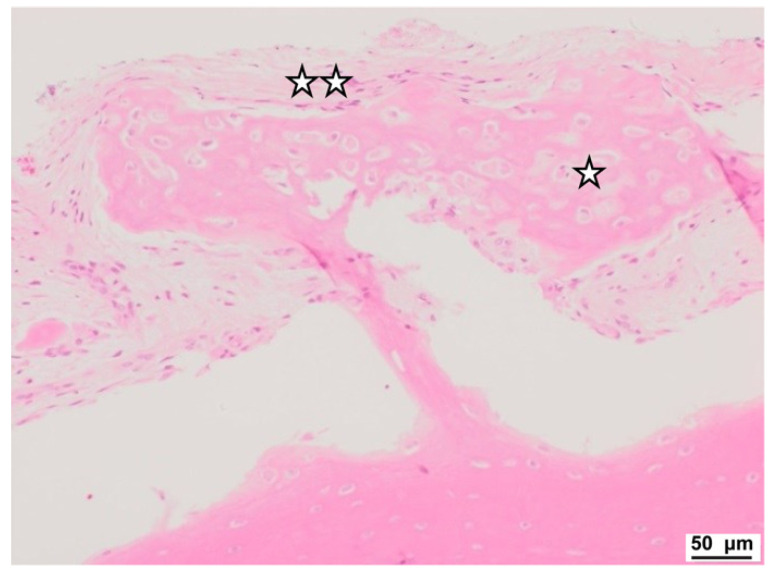
Light microscopy image. Regenerative processes with undifferentiated trabeculae. Bone structures (☆), numerous osteoblasts are present (☆☆). Gap is artificial due to cutting process. Hematoxylin and eosin staining.

**Figure 5:**
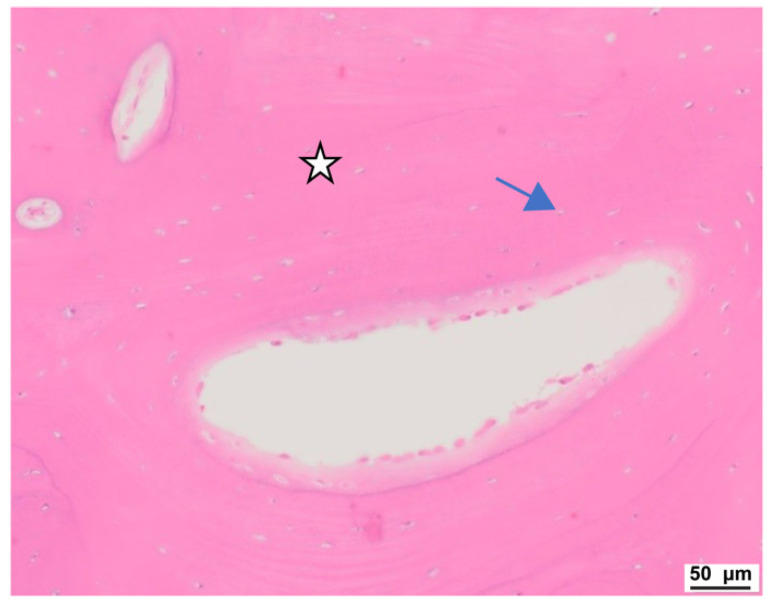
Light microscopy image. Lamellar structure of the bone is present (☆). Osteoblasts can be seen (arrow). Hematoxylin and eosin staining.

Histological analysis showed a dense outer bone layer with well-differentiated, clearly defined lamellae and organized osteocyte distribution. Vascularization appeared normal, though some capillary vessels displayed signs of hyperemia, possibly indicating active remodeling. Van Gieson’s staining highlighted the structured arrangement of lamellae, with regional variations in collagen content and bone regeneration, suggesting areas of active remodeling and maturation (Figure 4 and Figure 5).

#### 4.1.3. Electron Microscopy Results

**Figure 6:**
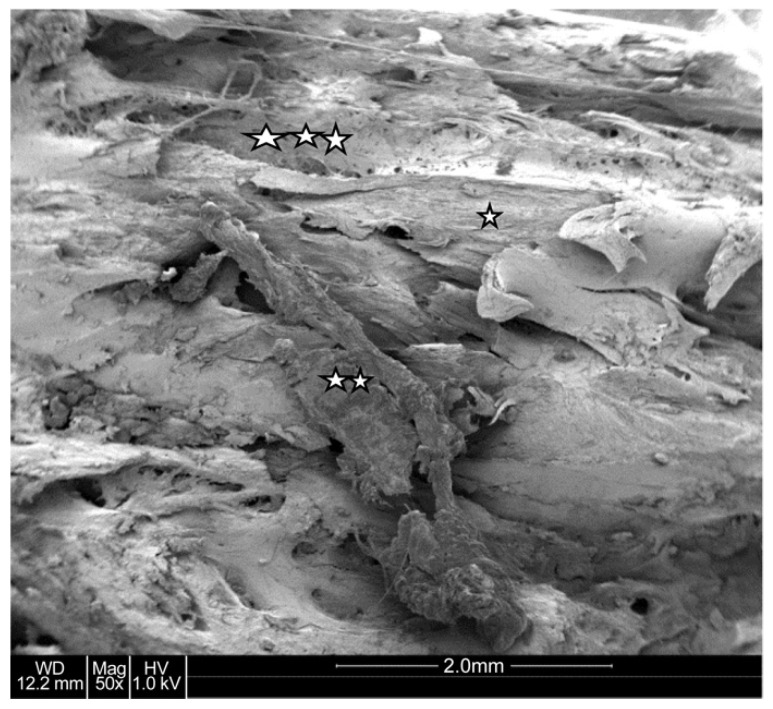
Scanning electron microscopy image. Characteristic view of a zone with compact bone (☆). Growing osteoblasts (☆☆). Vessels signify vascularization (☆☆☆). No collagen fibers.

**Figure 7:**
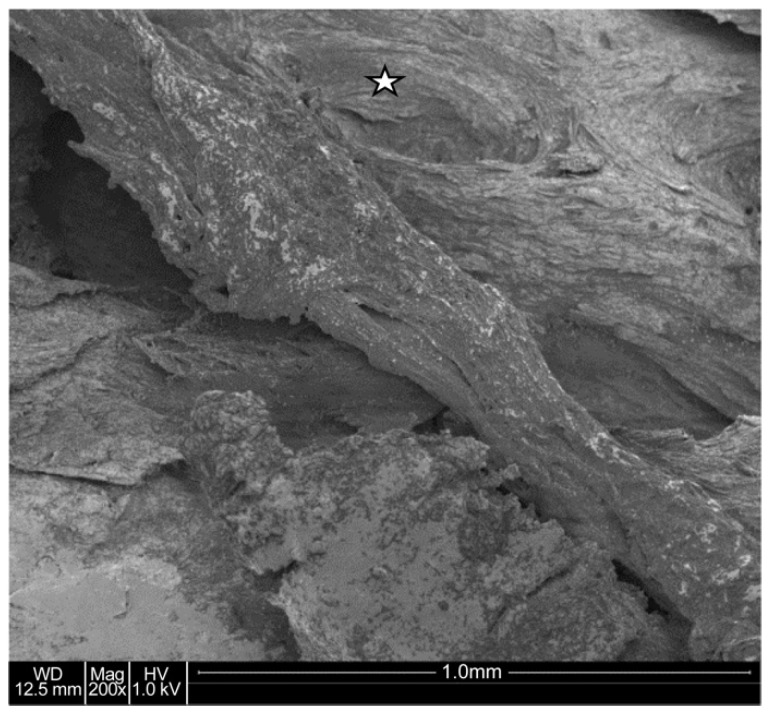
Scanning electron microscopic image. The lamellar structure of the bone is obvious (☆); compare with Figure 5.

**Figure 8:**
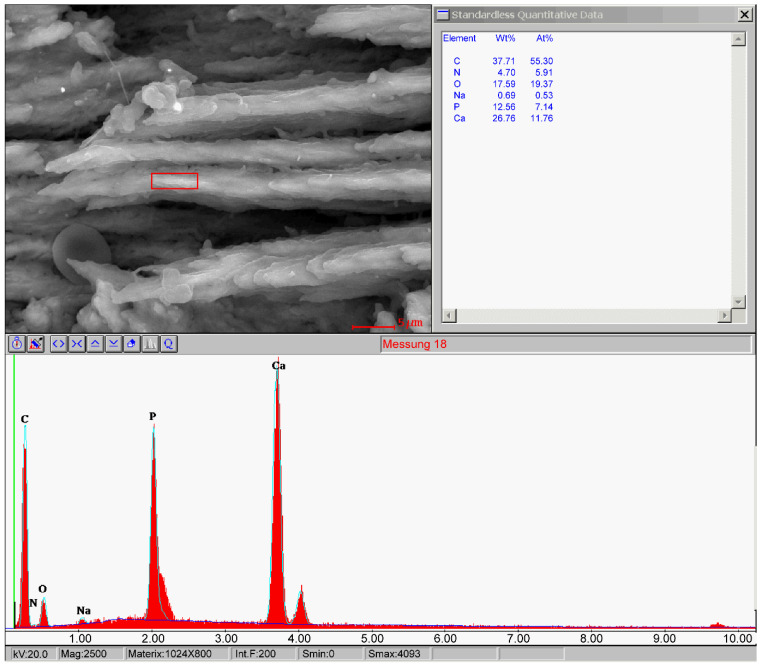
Scanning electron microscopy image of compact new build bone. EDS analysis was used to determine elemental composition. Ratio of Ca/P is 1.65:1.

Scanning electron microscopy provided a highly detailed view of the bone’s microarchitecture. The images revealed a stratified arrangement of polygonal bone cells, with visible osteoblasts indicating active bone formation. The surfaces of the newly formed bone were well-vascularized, contributing to the bone’s strength and stability (Figure 6 and Figure 7). Energy-dispersive microanalysis showed a Ca/P ratio of 1:1.65, consistent with the normal mineral composition of bone (Figure 8).

## 5. Discussion

Bilateral cosmetic limb lengthening is becoming more popular. Since healthy individuals undergo this surgery, a statement about the quality of the newly regenerated bone is of huge interest. To our knowledge, and following an intensive literature review, we describe the first case with a specific analysis of human regenerated bone (with a significant length of the regenerated bone) harvested after primary leg lengthening and subsequent shortening in the further course. Although extensive animal experiments have improved our understanding of the radiological, histological, and pathophysiological processes during DO, they have never been studied in a living human being.

Some studies [24,25,26,27,28,29,30,31,32,33] have previously reported that DO has features of embryonic, fetal growth, neonatal limb development, and normal fracture healing [4,13]. In a review, Choi et al. discussed the radiographic and histologic features of DO [14]. However, the exact cellular and molecular mechanisms of osseous and non-osseous regeneration are yet to be investigated.

In our study, we found that high-resolution computed tomography of the harvested samples showed all features characteristic of healthy bone (lamellar structure, trabeculae, canaliculi, marrow canal, and dense compact bone layer). At 1128 HU and 1247 HU, respectively, bone density was comparable to that of normal adult bone > 500–1900 HU [15,16].

In contrast to fracture healing, in DO, bone formation is primarily intramembranous ossification [17,18]. Early fracture healing starts with hematoma formation and the recruitment of inflammatory cells and stem cells [18,19]. Jazrawi et al. [20] described early evidence of endochondral bone formation, although no cartilage was observed within the distraction gap. In histologic analysis of our specimens, no chondrocytes were present. This finding supports the assumption of Jazrawi et al. that a distracting environment might suppress cartilage development [18,20]. The bone architecture was comparable to that of healthy adult bone with a well-defined compact bone layer consisting of partially dense regular lamellae. Furthermore, we found regular vascularization and distribution patterns of osteocytes. This too is comparable to normal bone [18,19,20].

As an indication for cell growth, in scanning electron microscopy, polygonal cells with stretching pods as well as osteoblasts were found. In energy-dispersive microanalysis, calcium and phosphate were shown to be present at a ratio of 1.65:1, which corresponds to that of normal bone [21].

However, unlike in adult bone, we found hyperemic capillaries. In 2002 Choi et al. stated that angiogenesis is much greater in DO than in fracture healing [14,19]. This may be attributed to the fact that, in fracture healing, angiogenesis begins 1–2 weeks after the trauma; however, in the case of DO, proof of angiogenesis is displayed [18,19,22].

This is the first time human bone has been analyzed after DO. Our patient has demonstrated that while limb lengthening by 12 cm poses challenges, the human body is biologically capable of overcoming them. Our findings support the commonly accepted theory that bone is unique regarding its healing without the formation of scar tissue.

Lengthening with intramedullary nails, such as the Fitbone (Orthofix, Lewisville, TX, USA), Betzbone (Medi-Tech, Wadern, Germany), PRECICE nail (NuVasive, San Diego, CA, USA), and other magnetic lengthening nails (MLN), has emerged as a promising alternative to traditional external fixation methods for bone lengthening and deformity correction. However, despite the advantages of these techniques, various challenges and complications have been reported in the literature. One of the major drawbacks of nail lengthening is the increased risk of requiring additional surgical procedures. Frommer et al. [28] noted that patients who underwent femoral lengthening with magnetically driven antegrade intramedullary nails often required multiple surgeries as part of their treatment. Many patients also experienced temporary joint stiffness, which negatively impacted their recovery and final outcomes.

Another challenge frequently discussed is the prolonged time to bone union associated with MLN compared to other techniques, such as lengthening and then nailing (LATN). Dvorzhinskiy et al. [26] observed that although MLN required fewer procedures than LATN, it resulted in a longer time for bone union following tibial lengthening. This extended healing period can lead to increased discomfort and a longer recovery time for patients.

Using magnetic expandable intramedullary nails for femoral lengthening in skeletally immature patients also presents unique challenges. Dahl et al. [29] emphasized the need for careful planning of implant length, preventing knee subluxation during elongation, and minimizing deformity within the regenerated tissue. Future internal lengthening technologies should be specifically designed to ensure safe limb lengthening in this age group, as most current devices are used off-label in younger patients.

Cost is another important factor when comparing lengthening nails to other methods. Richardson et al. [27] found that the average cost for femoral lengthening using MLN and lengthening over nail (LON) was comparable, though the higher implant cost of MLN must be considered. However, this increased cost may be offset by the reduced number of procedures required with MLN. Other factors, such as the patient’s age and the presence of a visible periosteal callus, have been found to influence bone healing outcomes during distraction osteogenesis, as noted by Jochymek et al. [37,38]. This highlights the complexity of the bone healing process during distraction osteogenesis and underscores the need for further research to identify factors that can optimize outcomes.

Despite our findings, no conclusions should be made regarding biomechanical features during dynamic cyclic loading, since no biomechanical testing was performed in this case. Additionally, from what we have seen, some cautions and suggestions can be given. Our case report shows that it is very important to choose patients carefully and plan thoroughly before the operation for nail DO. We should also take care to monitor consistently after surgery, with a focus on proper bone healing as well as being ready to identify and handle complications if they happen. Thus, we suggest more research with increased sample sizes and longer periods of follow-up to comprehend the results in the long run for DO. Additionally, future investigations should look into the mechanical qualities of regenerated bone, which would help in obtaining a complete understanding of the newly formed bone’s quality.

## 6. Conclusions

Radiological, histological, and microscopic examination of harvested bone after DO reveals a high-quality bone structure comparable to that of healthy native human adult bone tissue. This proves that bone is a unique tissue, healing without scar formation. It regains its full strength, elasticity, and function.

## Data Availability

Data is available on request from the corresponding authors.
